# Transboundary nomadic population movement: a potential for import-export of poliovirus

**DOI:** 10.1186/s12889-018-6190-2

**Published:** 2018-12-13

**Authors:** Samuel Bawa, Mojisola Afolabi, Khalid Abdelrahim, Goni Abba, Adamu Ningi, Salome Yakubu Tafida, Sisay G. Tegegne, Charity Warigon, Terna Nomhwange, Sadiq Abubakar Umar, Aron Aregay, Ahmed Fanti, Bakoji Ahmed, Peter Nsubuga, Usman Adamu, Fiona Braka, Alemu Wondimagegnehu, Faisal Shuaib

**Affiliations:** 1 World Health Organization, Country Representative Office, Abuja, Nigeria; 2 World Health Organization, Bauchi State Office, Bauchi, Nigeria; 3 Federal Ministry of Agriculture, Abuja, Nigeria; 4 World Health Organization, Intercountry Support Team, Harare, Zimbabwe; 5 Bauchi state Primary Health Care Development Agency, Bauchi, Nigeria; 6 Global Public Health Solutions, Atlanta, GA USA; 7 National Primary Health Care Development Agency, Abuja, Nigeria

**Keywords:** Nomadic population, Transboundary, Poliomyelitis, One-health

## Abstract

**Background:**

Nomadic populations have a considerably higher risk of contracting a number of diseases but, despite the magnitude of the public health risks involved, they are mostly underserved with few health policies or plans to target them. Nomadic population movements are shown to be a niche for the transmission of diseases, including poliomyelitis. The nomadic routes traverse the northern states of Nigeria to other countries in the Lake Chad subregion. As part of the February 2016 polio supplemental immunization activity (SIA) plans in Bauchi state, a review of nomadic routes and populations identified a nomadic population who originated from outside the international borders of Nigeria. This study describes the engagement process for a transboundary nomadic population and the interventions provided to improve population immunity among them while traversing through Nigeria.

**Methods:**

This was an intervention study which involved a cross-sectional mixed-method (quantitative and qualitative) survey. Information was collected on the nomadic pastoralists entry and exit points, resting points, and health-seeking behavior using key informant interviews and semistructured questionnaire. Transit vaccination teams targeted the groups with oral polio vaccines (OPVs) and other routine antigens along identified routes during the months of February to April 2016. Mobile health teams provided immunization and other child and maternal health survival interventions.

**Results:**

A total of 2015 children aged under 5 years were vaccinated with OPV, of which 264 (13.1%) were zero-dose during the February 2016 SIAs while, in the March immunization plus days (IPDs), 1864 were immunized of which 211 (11.0%) were zero-dose. A total of 296 children aged under 1 year old were given the first dose of pentavalent vaccine (penta 1), while 119 received the third dose (penta 3), giving a dropout rate of 59.8%.

**Conclusions:**

Nomadic pastoralists move across international borders and there is a need for transboundary policies among the countries in the Lake Chad region to improve population immunity and disease surveillance through a holistic approach using the One-health concept.

## Background

Nomads and migrant populations are one of the most underserved communities regarding health and social services worldwide. Health indicators suggest that nomads are at considerably higher risk of contracting a number of diseases but, despite the magnitude of the public health risks involved, there are few health policies or plans to target them [1, 2].

The trajectory of the nomadic pastoralist population often traverses transnational borders, and these movements are shown to be a niche for the transmission of diseases [3, 4]. Detailed polio outbreak investigation reports indicate that up to 40% of cases of wild poliovirus (WPV) isolated in the Republic of Chad were among nomads [5]. Reports from India also show that large migrant subpopulation movements are important contributors to WPV transmission [6, 7]. Furthermore, findings from Ethiopia show a risk of transnational transmission of WPV among nomadic pastoralists [8].

The 2014 Nigeria polio eradication emergency plan (NPEEP) acknowledged that the movement of nomadic populations across the country posed a risk for the spread of the circulating virus with a risk of re-infecting other states. There are many nomadic routes traversing the northern states of the country, in all directions, in search of pasture and favorable climes agreeable to their vocation [9]. However, some of the routes traverse other countries in the Lake Chad subregion.

The strategic priorities identified by the NPEEP include reaching children in underserved nomadic populations [9].

During the planning and review of nomadic routes and populations for the February 2016 supplemental immunization activities (SIAs) in Bauchi state, a nomadic pastoralist population who originated from Niger Republic and passed into Nigeria through its international borders were identified.

This study describes the process of the engagement of this transboundary nomadic population, their movement pattern, health-seeking behavior, and the interventions provided to improve the population immunity against childhood killer diseases such as poliomyelitis, measles, pertussis, tuberculosis, diphtheria, and tetanus while migrating in Bauchi state, Nigeria.

## Methods

As part of the process for conducting SIAs in Nigeria, the micro-planning process is followed to identify areas, characterize the population, and quantify and identify resource requirements. This process was conducted as itemized below.

### Identification and mapping of local government areas (LGAs) with nomadic populations and community engagement

In preparation for the exercise in February2016, the entry and exit routes, resting/watering points, and grazing areas of nomadic populations were reviewed in Bauchi state and the affected local government areas, wards, and potential camps were identified. This is part of the analysis for identifying potential areas and high-risk populations for polio transmission and developing strategies to target them.

We made contact with the leadership of the nomads, mobilized them, and identified focal persons among them for easier contact and planning of immunization activities to target them. Furthermore, indigenous community leaders in the settlements closest to where they were found were also used as an entry point. They were sensitized and very receptive once it was explained to them the benefit of immunization and other health interventions that their eligible children and women would have.

### Rapid appraisal of movement pattern and health-seeking behavior

Rapid collection of information from the nomads was exigent, and a key informant interview was used to rapidly appraise their pattern of movement and their health needs. We collected information on the routes (dynamics of movement), knowledge, attitude, and health-seeking behavior of the nomadic and migrant population. A total of 120 key informant interviews were conducted (two per ward) using a semistructured instrument with questions asked according to thematic areas of health-seeking behavior, pattern of movement, healthcare access, and utilization of the nomads and their herd. The information collected was analyzed and used to develop plans for implementation of the vaccination exercise.

### Interventions (strategies to reach the nomadic population)

Transit vaccination teams were trained to administer oral polio vaccines (OPVs) depending on the size of the nomadic population and the routes identified during the micro-planning process. At least 2–5 teams per ward were deployed and accompanied by local interpreters. Senior supervisors from the LGAs and supported by partners were deployed for the exercise that lasted for 3–4 days during the days of each round of February and March 2016 SIAs. Furthermore, routine immunization antigens and treatment of minor ailments were offered in some LGAs.

In addition, and in between the SIA campaigns, mobile health teams targeted them to provide immunization and other child and maternal health survival interventions in the form of vitamin A supplements, deworming tablets, treatment of minor ailments, and health promotion.

### Data collection and analysis

We collected information on the pastoralist transit and resting points during the course of their movement. Polio, routine immunization, and treatment coverage data were collected and percentages derived from them. The transit and resting points of the pastoralists were also transposed onto the regional map. This project was not intended as research work, but instead as an intervention to improve vaccination uptake among nomads and, as such, ethical clearance was not required. However, the government of Bauchi state granted permission for the intervention.

## Results

**Fig. 1:**
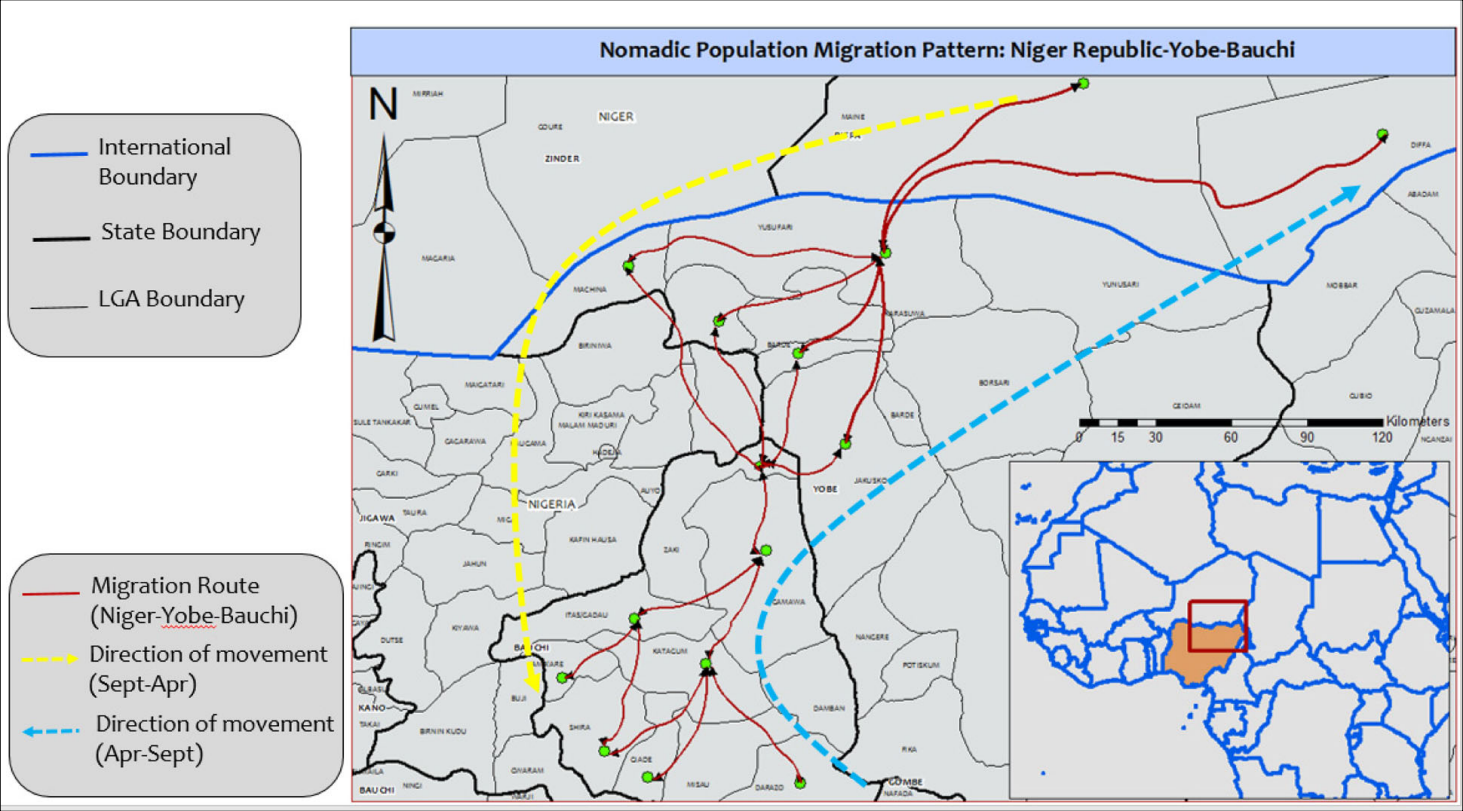
Map, showing the migration pattern of transboundary nomads; Niger Republic; Yobe–Bauchi States/LGAs, Nigeria

The information collected from the respondents showed that they enter Nigeria from Niger Republic in batches in September and October and do not go back to until April/May of the following year. They cross the Niger-Nigeria border and settle in the border LGAs of Yusufari, Nguru, Machina, Jakusko, and Bade in Yobe state, before moving into Zaki settlement (Gumai and Tashena wards) in Bauchi state. Zaki LGA is their main point of entry into Bauchi state and they move from there to other LGAs, namely Gamawa, Itas Gadau, Darazo, Katagum, Shira, Giade, and Jama’are. The return trip back starts in April/May 2016; they would settle again in the listed four LGAs in Yobe State before finally crossing the border into Niger Republic in July/August 2016 (Fig. 1). These nomads mainly originate from Maine-Soroa District, Kilakam, Kanguri/Kilori/Affau-Shatimanu of Diffa Region in the southeastern Niger Republic. According to one of the respondents, Alhaji Sule Muhammad, “this has been our pattern of movement for over 30 years”. However, they also reported that some of their group members come into Nigeria through Yobe and Borno states, but their movement in that direction has been affected by insurgent activities in recent years. The main purpose of their movement is the grazing of their animals (camels) and to sell the milk (believed to be medicinal) and potash.

Respiratory tract infections, malaria, measles, febrile illness, cough, cold, diarrhea, and skin diseases are found to be the common conditions/ailments affecting children, women, and men. Although they attended a health facility along with their routes, they also used traditional medications, prayers, and consumption of camel milk and urine, which are believed to be medicinal.

Their knowledge on polio immunization and protection against other vaccine-preventable diseases was low (25%) among the respondents. They could mention few diseases that can be prevented through vaccinations, and they were aware of the polio supplemental campaigns. Almost 60% reported that their eligible children had received at least one dose of OPV and had received most of their sources of information on polio from the radio (100%), traditional leaders (75%), health workers (50%), and friends/relations (25%). However, knowledge of the reporting of acute flaccid paralysis (AFP) and other diseases was very poor. Most of the respondents were aware that malaria could be prevented by sleeping under insecticide-treated nets, especially for the children and women.

The target population of nomads determined through the micro-planning process varied in location in the months of February and March 2016. The locations of camps in Darazo LGA were deserted; the population of camps increased in Gamawa, Shira, Zaki, and Itas Gadau, but decreased in Giade and Katagum in March 2016 (Table 1).

**Table 1:** Distribution of camps and target population of nomads; February and March immunization plus days (IPDs), 2016, Bauchi state

LGA	February 2016 IPDs			March 2016 IPDs		
	No. of wards	No. of camps	< 5 years of age target population	No of wards	No. of camps	< 5 years of age target population
Gamawa	7	10	251	8	11	420
Giade	8	13	83	6	9	59
Shira	7	10	158	6	10	184
Zaki	9	32	417	13	53	528
Darazo	2	3	271	0	0	0
Katagum	11	21	661	11	17	251
Itas Gadau	16	35	587	12	36	594
Total	60	124	2428	56	136	2036

*LGA* Local Government Area

During the February 2016 round of immunization plus days (IPDs), a total of 2015 eligible children were vaccinated with OPV, out of which 264 were zero-dose (eligible children who have never received any OPV dose) representing 13.1% of the total immunized. During March IPDs, 1864 were vaccinated out of which 211 were zero-dose, representing 11% of the total immunized. The camps earlier identified and targeted during the February IPDs in Darazo did not have any inhabitants during the March 2016 IPDs as the nomads had changed location (Table 2).

**Table 2:** Children vaccinated with oral polio vaccine in nomadic camps; February and March immunization plus days (IPDs), 2016, Bauchi state

LGA	February 2016 IPDs				March 2016 IPDs			
	Zero-dose	Other doses	Total	% zero-dose	Zero-dose	Other doses	Total	% zero-dose
Gamawa	71	178	249	28.5	1	430	431	0.2
Giade	1	78	79	1.3	3	58	62	5.2
Shira	8	190	198	4.0	5	219	224	2.3
Zaki	3	432	435	0.7	78	324	402	24.1
Darazo	6	86	92	6.5	0	0	0	0.0
Katagum	81	373	454	17.8	32	226	258	14.2
Itas Gadau	94	414	508	18.5	93	394	487	23.6
Total	264	1751	2015	13.1	211	1651	1864	12.8

*LGA* Local Government Area

Children under 1 year old were provided with other routine immunization antigens. A total of 296 children were given the first dose of pentavalent vaccine (penta 1) while 119 received up to the third dose of pentavalent vaccine (penta 3), giving a dropout rate of 59.8%. Treatment of minor ailments was provided to 1243 patients (Table 3).

**Table 3:** Routine immunization antigens, vitamin A, deworming, and treatment of minor ailments provided to the nomadic population February to April 2016, in Local Government Areas (LGAs) of Bauchi state

LGA	Penta 1 (< 1 year)	Penta 2 (< 1 year)	Penta 3 (< 1 year)	Measles (9–11 months)	Vitamin A administered		Albendazole	Treatment of minor ailments	
					6–11 months	12–59 months	12–59 months	Patients seen	Referrals
Dambam	12	10	3	24	8	59	59	55	0
Gamawa	133	87	84	111	161	877	898	532	8
Itas Gadau	30	16	0	28	49	151	328	100	0
Shira	91	46	16	62	57	471	475	461	70
Zaki	30	21	16	105	36	100	100	95	0
Total	296	180	119	330	311	1658	1860	1243	78

Penta 3 dropout rate = 59.8%

## Discussion

We found that the nomadic population was constantly moving, and efforts to meet their health needs required continuous tracking and interaction with the local population and wide stakeholder involvement. In a review of published literature, Gushulak et al. [10] reported that international population mobility is an underlying factor in the emergence of public health threats and risks that must be managed globally.

The Polio eradication initiative (PEI) team tracked and provided vaccination and other health interventions to a group of transboundary nomads who move between southern Niger Republic and northeastern Nigeria. There was a high proportion of children receiving OPVs among the nomads but also a high dropout of the children from the routine immunization schedule. This high dropout is similar to findings from previous studies in Nigeria, which showed that the migration status of the mothers affects the likelihood of their child receiving full immunization [11]. It also corroborates with the findings from small and localized studies which suggested very low vaccination coverage, with no fully immunized nomadic children [5]. The study estimated coverage of the third doses of diphtheria, tetanus, pertussis, and polio vaccines (DPT3/OPV) among nomadic children aged 0–11 months to be 8% in 2003 and 14% in 2004 in the subdistrict of Gredaya, Chad. Furthermore, studies on cross-border WPV transmission in Ethiopia showed a poor knowledge of vaccine-preventable diseases and of the need to report cases of AFP and outbreak of diseases among nomads. The immunization coverage was also low, and mechanisms for prevention of cross-border polio transmission was almost nonexistent [8]. Conversely, mobile phones were used for surveillance of the mobile pastoralist camps to provide usable, valid information which would likely lead to improved planning and provision of human and animal healthcare [12].

The nomads had concerns for disease conditions that affected their children, although they use local and inherited traditional methods for managing these conditions.

Despite the large number of animal populations in Nigeria, there were no systematic plans to engage with the transboundary nomads by either the animal or human health sectors, especially to provide vaccinations or other forms of healthcare for them and their animals.

Innovative and integrated health services for nomads have been developed due to the nomadic pastoralists sharing a common and similar way of life, driven by the needs of their animals. These services for nomads were provided in different settings with positive and synergistic results [13–15].

The risk of transboundary transmission of polio and other infectious diseases remains high. Countries should target special populations (for example, the nomadic pastoralist) using inter-sectoral collaboration to develop and strengthen holistic policy on their health and social development.

Nomadic pastoralists move across international borders. There is the need for transboundary regional initiatives among the countries in the Lake Chad region to improve population immunity and transborder disease surveillance, and to ensure effective control and prevention of diseases within the One-health concept.

## Conclusions

Nomadic pastoralists are a dynamic population that move across international borders with a high risk of transboundary disease. The polio eradication program was planned to target the nomadic population and provide vaccination and other child and maternal health services in the course of their movement. Hence, there is a need for transboundary policies among the countries in the Lake Chad region to improve population immunity and disease surveillance through a holistic approach using the One-health concept.

## Abbreviations

AFP: Acute flaccid paralysis
IPD: Immunization plus day
LGA: Local Government Area
NPEEP: Nigeria polio eradication emergency plan
OPV: Oral polio vaccine
PEI: Polio eradication initiative
SIA: Supplemental immunization activity
WPV: Wild poliovirus
